# Structured Professional Judgment to Assist the Evaluation and Safety Planning of Suicide Risk: The Risk of Suicide Protocol (RoSP)

**DOI:** 10.3389/fpsyt.2021.607120

**Published:** 2021-05-17

**Authors:** Nicola S. Gray, Ann John, Aimee McKinnon, Stephanie Raybould, James Knowles, Robert J. Snowden

**Affiliations:** ^1^Department of Psychology, Swansea University, Swansea, United Kingdom; ^2^Swansea University Medical School, Swansea, United Kingdom; ^3^Oxford Institute for Clinical Psychology Training and Research, University of Oxford, Oxford, United Kingdom; ^4^Independent Researcher, Cardiff, United Kingdom; ^5^School of Psychology, Cardiff University, Cardiff, United Kingdom

**Keywords:** suicide, self-injury, self-harm, structured professional judgement, risk management

## Abstract

**Background:** The Risk of Suicide Protocol (RoSP) is a structured professional judgment (SPJ) scheme designed in line with NICE guidelines to improve clinicians' ability to evaluate and manage suicide risk.

**Aims:** This study aimed to evaluate the efficacy of RoSP in two settings: (1) unexpected deaths of people in the community who were known to mental health services; and (2) an inpatient hospital specializing in the assessment and treatment of patients with personality disorder.

**Method:** In Study 1, information from a database of unexpected deaths (*N* = 68) within an NHS health board was used to complete a RoSP assessment (blind to cause of death) and information from the Coroner's Court was used to assign people to suicide vs. natural causes/accidental death. In Study 2, patients (*N* = 62) were assessed on the RoSP upon admission to hospital and their self-injurious behaviors were recorded over the first 3 months of admission.

**Results:** (1) Evaluations using RoSP were highly reliable in both samples (ICCs 0.93–0.98); (2) professional judgment based on the RoSP was predictive of completed suicide in the community sample (AUC = 0.83) and; (3) was predictive of both suicide attempts (AUC = 0.81) and all self-injurious behaviors (AUC = 0.80) for the inpatient sample.

**Conclusion:** RoSP is a reliable and valid instrument for the structured clinical evaluation of suicide risk for use in inpatient psychiatric services and in community mental health services. RoSP's efficacy is comparable to well-established structured professional judgment instruments designed to predict other risk behavior (e.g., HCR-20 and the prediction of violence). The use of RoSP for the clinical evaluation of suicide risk and safety-planning provides a structure for meeting NICE guidelines for suicide prevention and is now evidence-based.

## Introduction

In the UK suicide is the leading cause of death in males aged 5–49 and in females age 5–34 (1). In the USA suicide is the 10th most common cause of death, the 2nd most common in 10–34 year olds (2), and leads to an estimated $51 billion in combined medical and work loss costs (3) with similar rankings in other developed regions worldwide (4, 5). Providing effective risk assessment, support and safety planning for those at risk of suicide is of paramount importance and poses a major challenge to professionals and health care services since suicidal ideation and suicide attempts are relatively common and death by suicide is relatively rare (6). Past research has challenged the predictive accuracy of unaided clinical judgements across a range of human risk behaviors (7) and previous studies have demonstrated that global clinical judgements of future self-injurious and suicide risk by psychiatric staff have low predictive value (8). A number of statistical or actuarial scales have arisen in an effort to help clinicians assess and manage suicide risk. However, these tools often fail to predict accurately future suicidal and self-injurious behaviors (9–11) and there is a concern that such measures place too much emphasis on labeling levels of risk and not enough emphasis on understanding and managing the risk. These problems have led to the National Institute for Health and Care (NICE) giving guidance against the sole use of actuarial “tick-box” risk assessment instruments. In particular, NICE guidelines (12) explicitly state: “do not use risk assessment tools and scales to predict future suicide or repetition of self-harm,” (1.3.11 p.21) and “do not use risk assessment tools and scales to determine who should and should not be offered treatment or who should be discharged” (1.3.12 p.21).

In the domain of the assessment of violence to others in forensic psychiatric services, the debate between clinical opinion and actuarial instruments led to the development of the “Structured Professional Judgement” (SPJ) approach. SPJs are designed to systematically guide and assist a clinician in the production of a clinical formulation and a risk management plan (13) and are now regarded by many as the gold-standard method of violence risk assessment (14). Importantly, SPJs are not a form of actuarial instrument, where the focus is solely on obtaining a label of the level of risk of an individual, but are instead focused on structuring the clinician's assessment in a standardized and evidenced-based manner, and ensuring that all relevant areas of clinical risk are systematically assessed. There has been one previous attempt to develop an SPJ tool designed for the assessment of suicide risk (15). The Suicide Risk Assessment and Management Manual (S-RAMM) is a SPJ tool that has demonstrated good inter-rater reliability (16) and a good ability to prospectively predict self-injurious and suicidal behavior (17). However, despite the S-RAMM being developed in 2003, with validation studies being published in 2009 (17), it has not received wide acceptance in clinical practice (18). The current research reports the initial validation of the Risk of Suicide Protocol (RoSP). The RoSP is an SPJ scheme predicated upon the methodology and structure of the HCR-20 for violence risk assessment (19) and designed to facilitate detailed evaluation and safety planning of suicide risk with the development of an associated individualized risk management plan that meets NICE recommendations.

Whilst there is overlap between these two SPJ approaches to suicide evaluation, there are a few key differences between the two assessments. Firstly, the S-RAMM contains three subscales (Historical Factors, Current Clinical Factors, and Future Clinical Factors), whereas the RoSP comprises of four subscales (History, Current Clinical, Current Crisis, and Current Thinking). When considering the individual risk factors included in the two assessments, the RoSP places more emphasis on the individual's current social circumstances. Whilst the S-RAMM has one item (Psychosocial Stress) dedicated to evaluating the individual's current social circumstances, the RoSP has five items dedicated to considering an individual's physical health, romantic relationships, employment or financial situation, whether they are experiencing difficulties with the law, and whether they have experienced a recent loss in their life. These five risk factors were designed to structure a clinician's standardized assessment of current psychosocial difficulties and the interaction between these and their mental health. The added emphasis placed on social factors was designed to ensure that the RoSP adhered closely to the NICE guidelines that suggest the main components of an assessment of need following an episode of self-injury should include the individual's physical health issues, social circumstances and problems, personal relationships, financial problems and recent life difficulties (12). Moreover, whilst the S-RAMM contains items relating to demographic risk factors (e.g., age, gender, marital status), the RoSP deliberately excludes such items in an effort to focus more on dynamic risk factors that can be targeted and ameliorated through treatment and management. The RoSP aims to move away from a focus exclusively upon any variable with predictive validity (as would be contained in an actuarial risk instrument) to modifiable risk factors which clinicians can work upon to try to reduce and manage suicide risk moving forward. For example, although different age groups are associated with different rates of suicidal behavior (2), a clinician could not reasonably suggest altering one's age as a potential treatment or management plan. The same argument applies to gender.

An additional difference between the two measures is that the RoSP asks clinicians to consider an individual's history of violent behavior and their current feelings of anger. These items were included in the RoSP due to past research demonstrating that historical violence is associated with a 5-fold increase in risk of death from suicide (20) and that feelings of anger and hostility are linked to increased risk of suicide attempts (21, 22). Thus, it was felt important that an evaluation of a person's anger and risk behavior to others (in terms of past violence and aggression) was incorporated into the formulation about risk to self. Finally, the RoSP (20 items) contains fewer items than the S-RAMM (23 items). This is likely to be an important factor when considering the palatability of these assessments for clinical staff working in time-pressured clinical environments.

In summary, the Risk of Suicide Protocol (RoSP) is an SPJ scheme designed to facilitate detailed evaluation and safety planning of suicide risk with the development of an associated individualized risk management and treatment plan that meets NICE recommendations. In this paper we evaluate the efficacy of the RoSP in two different populations: (1) a community setting for patients known to mental health services (a retrospective study); and (2) an in-patient setting for patients with a personality disorder (a prospective study).

## Method

For study 1, the RoSP was evaluated by trained RoSP raters blind to the cause of death, from multi-disciplinary mental health records available at the last known contact with mental health services. These records were “cleaned” by another person who was not a rater to ensure that there was no mention of the cause of death contained therein. Data were entered into an anonymized database for research purposes. Ethical permission for the study was given by the National Health Service Research Ethics Committee (15/EM/0044).

For study 2, the RoSP was used as part of the standard assessment for patients as they were admitted to the hospital. Data on behavior, including all episodes of self-injury were recorded on a daily basis by nursing staff. Permission to use an anonymized version of this clinical database for research was granted by the National Health Service Research Ethics Committee (14/EM/1178).

### Participants

For Study 1, information was gathered on service users who were known to mental health services in a UK NHS Health Board who had died unexpectedly between March 2009 and March 2013. Based on the verdict of an inquest in a Coroner's Court, two groups were defined: one where the Coroner judged the person to have completed suicide or had recorded an open verdict (suicide group), and one where the Coroner judged the person to have died from natural causes or from an accidental death (non-suicide group). People within the Older Adult Services were excluded from the study as it was decided that these people would be more likely than the other groups to have died from natural causes and this would have led to a systematic bias. Cases where the Coroner's verdict was unknown or uncertain were eliminated from the study. The suicide group consisted of 39 (57.9% male) cases and the non-suicide group consisted of 29 (58.6% male) cases. Mean ages were 50.1 and 45.1 years, respectively, which did not differ significantly [*t*(65) = 1.78, ns]. No other demographic details were recorded that were not otherwise coded within the RoSP (i.e., psychiatric diagnosis is included in the RoSP evaluation).

For study 2 participants were in-patients, resident in a low secure unit (Ty Catrin, Pastoral Healthcare, Cardiff) that specializes in the treatment and management of service users with a diagnosis of a personality disorder and who were admitted to the psychiatric hospital over the period of 2009–2014. The patients constitute a high-risk group as they were detained under the UK Mental Health Act (1983) due to the risk they posed to self and/or others. The hospital completed the RoSP as part of the initial clinical assessment of all service users. The assessments were completed within 1 week of admission by the service user's clinical psychologist (who was fully trained in RoSP) using both past clinical records and clinical interviews. This initial RoSP would be regularly updated according to new information (including new acts of self-injury) and treatment response in order to evaluate dynamic risk to self, as per recommended clinical practice. However, the present analysis uses only the first completion of the RoSP.

Of the 68 patients admitted to the hospital during the period of study, a RoSP was completed on 62 [36 women: mean age 30.0 (*SD* = 8.9, Range 18–56 years), 26 men: mean age 32.9 (*SD* = 12.6, Range 18–65 years)]. A RoSP was not completed on 6 patients because they were discharged from the hospital before a RoSP could be completed. Data for this study was taken from the first 3 months of the service user's stay at the hospital. All 62 patients stayed within the hospital for at least this period of time.

### Measures

#### Risk of Suicide Protocol—RoSP (Snowden and Gray, 2020)

The RoSP (version 1.0) was written in 2007. Whilst a review of the scientific literature was our main source for deciding what risk factors would constitute the RoSP. We also carefully considered the clinical utility of each item from both the point of view of the ease of obtaining information for the assessing clinician and whether the risk factor was “dynamic” in that it could be targeted for treatment or management. We deliberately chose risk factors that had these properties.

During the first few years of development, RoSP underwent several updates and alterations based on feedback from clinicians and staff who were piloting the instrument in clinical practice. Items on the RoSP were rewritten or clarified, and some were dropped or morphed into the present version which we will refer to simply as RoSP. We mainly piloted the instrument in a low secure hospital for the assessment and treatment of people with personality disorder, as many of this group of service-users had complex clinical needs and posed many risks to both themselves and others. These complex clinical challenges afforded the developers of the RoSP a good opportunity to work through complex issues of assessment with the staff involved and also to have multiple outcome variables for analysis in what are relatively rare events in the general population (self-harm and attempted suicide). There were no completed suicides during the period of development of the RoSP, due mainly to the excellent and intensive clinical care that these patients were afforded by their clinical team.

The RoSP consists of 20 items that the clinician evaluates *before* formulating possible suicide risk of the individual and before making a judgement about the level and nature of safety planning and clinical intervention required for the service user, on the basis of suicide risk. Evaluation of each risk factor is not a simple “present” or “absent” as it would be for an actuarial instrument, but rather a detailed clinical formulation. The clinical formulation of each item attempts to provide details of the clinical presentation, or psychosocial problem, that is present for the person and to how the factor may be driving or maintaining suicide risk. Importantly, this includes not only clinical symptoms or presentation, but also psychosocial risk factors. An example of this would be for the item “Financial Problems.” On the RoSP we would not simply rate this as “present” or “absent,” but would provide detail of exactly how much debt, or financial difficulties, the person found themselves in and, crucially, their psychological or emotional reactions to this (e.g., feelings of failure, catastrophic thoughts about possible future outcomes, extreme anxiety about possible loss of home, loss of status, and loss off relationships, etc.). The preferred intervention would then be a psychosocial one—focusing on debt management—and perhaps on psychological intervention for the management of anxiety and catastrophic thoughts.

Thus, the process of completing the RoSP is designed to enhance safety planning for suicide and attempted suicide through the development of an individualized clinical formulation and risk management/treatment plan. We define suicide here as the deliberate attempt to take one's own life. We refer to acts of self-injury without the intention to die as “self-harm” and this is used synonymously with the term non-suicidal self-injury (NSSI), as used elsewhere in the literature (23). We acknowledge that this distinction between self-harm and attempted suicide is often difficult to ascertain (24) as it depends upon an evaluation of the person's intent at the material time, which is often unclear to the person themselves as well as to the clinical team providing care and management. We use the term “self-injury” to refer to any such act without regard for any intention to die or not.

The 20 items of the RoSP are separated into four domains (see Table 1). Detailed descriptions of the items, rationales for inclusion, and scoring guidelines are contained in the RoSP manual (obtainable on request from the first author). The first domain is termed “History” and evaluates historical factors, including previous suicide attempts and history of self-injury. The “Current^1^ Clinical” domain evaluates the clinical factors that are, or were, recently active (e.g., symptoms of depression, substance use problems). The “Current Crisis” scale evaluates current psychosocial stressors (e.g., loss of others, financial problems, legal problems), and the “Current Thinking” domain evaluates thinking style, including indications of suicidal ideation and intent, and feelings or thoughts of hopelessness and helplessness. We note that inclusion of these factors are consistent with NICE guidelines (12) that suggest that the main components of an assessment of need after an episode of self-injury should include the person's social situation (including current living arrangements, employment, and debt), personal relationships (including any recent breakdown of a significant relationship), recent life events, psychiatric history, and a mental state examination (including any history of previous self-injury and alcohol or drug abuse). Assessment should also include any enduring psychological characteristics that are known to be associated with self-injury and motivation for the act. Each of these areas of need, or risk, as set out in the RoSP are designed to be consistent with NICE guidelines and to assist the clinician to be adherent to these best practice guidelines. Each area of need is completed as a “mini-formulation,” presenting the details of each area of need for the person and how it has impacted on their mental state and ability to function, including the impact on the individual's suicidal ideation and intent. This process is then used to inform treatment and safety planning strategies specific to the individual.

**Table 1:** Items and structure of RoSP.

**History**
1. Suicide attempts
2. Self-injury
3. Violent behavior
4. History of major mental disorder
5. Membership of high risk group
**Current clinical**
6. Personality disorder
7. Current depressive symptoms
8. Substance use problems
9. Other current symptoms of mental illness
10. Poor treatment/management outcomes
**Current crisis**
11. Recent loss of significant other
12. Severe health problems
13. Relationship problems
14. Employment or financial problems
15. Problems with the law
**Current thinking**
16. Lack of personal support
17. Feelings of hopelessness
18. Feelings of anger
19. Suicidal ideation
20. Preparatory activity

The level of safety planning required for suicide risk was termed the structured professional judgment (SPJ) and could range from “*very low*,” to “*very high*” on a 5-point scale. For statistical purposes, each item was rated as present or absent by coding “Yes” if it was rated as being present or “No” if not present. If it was unclear as to whether an item was present or not, the item was rated as uncertain presence, or “?”. For usual clinical practice the pattern of presence or absence of risk factors would underpin the developing risk formulation (defined as how the pattern of the individual's risk factors and strengths interact to lead to an understanding of the “why” of suicide risk, thus dictating in turn the most effective risk management, or safety, plan).

For study 1, the RoSP evaluators (AMc, SR, and NSG) received training on the RoSP from the authors of this instrument. All RoSP evaluations were made blind to outcome (suicide vs. natural causes/accidental death). Completion of the RoSP was based on material available within the person's mental health records. The evaluation of “current” clinical status (i.e., in the recent past) was based on information within the mental health records taken at the time of the last clinical contact with mental health services prior to the person's death.

For study 2, ratings were based on collateral information (e.g., medical and psychiatric records) and clinical interview(s) with the service user. Clinical formulations using the RoSP were completed by the patient's allocated clinical psychologist (with the aid of psychology assistants) within the hospital, all of whom were trained on the use of SPJs in general and were RoSP trained.

### Aggression and Vulnerability Scale—AVS

The AVS (25) is a scheme for the evaluation and coding of problematic behaviors such as aggression to others and self-injury. Incidents were coded by clinical staff (nursing staff and psychology assistants trained in the use of the AVS) into one of 10 categories and a severity rating within this category was scored. The AVS has been shown to have good inter-rater reliability for both the categorical judgement and the severity judgment of behaviors (25).

### Analysis

For both studies, we used the structured clinical judgment derived from RoSP as our main measure, but also used the “scores” from the RoSP to look at the overall performance of the RoSP, the four subscales and each of the individual items (which we term RoSP scores, as for data analysis purposes it was necessary to assign numbers to the risk evaluation of each area of need). Each item on the RoSP was scored on a three-point scale. Participants scored “0” if that risk factor was absent, “1” if it was partially present, and a “2” if it was present. The total RoSP and subscales scores were calculated by summing the relevant item scores. Missing items were prorated. For study 1, we used a signal detection analysis and calculated the area under the curve (AUC) for the Receiver Operating Characteristic (ROC) for group membership.

For study 2, the data were analyzed in two complimentary ways. The data were recoded to reflect “present” or “not-present” for: (1) any self-injurious behavior; and (2) any suicide attempt for a ROC analysis. However, we also calculated correlations between the frequency of challenging behaviors and RoSP scores. Frequencies of self-injurious behaviors were highly skewed and therefore Spearman's rho was used.

## Results

### Study 1: Outpatients

#### Reliability

Ten cases (six men) were evaluated independently by two raters and the interclass correlation coefficients (ICCs) are shown in Table 2. All scales and subscales exhibited good to excellent reliability.

**Table 2:** Descriptive and inferential statistics for the RoSP in study 1.

	**Mean (SD)**	**Range**	**ICC**	**AUC (SE)**
				**Completed suicide**
SPJ	3.2 (1.2)	1–5	0.96^**^	0.83^**^ (0.05)
Total score	16.9 (6.1)	5–31	0.98^**^	0.80^**^ (0.06)
History	4.8 (2.3)	1–10	0.99^**^	0.63 (0.07)
Clinical	4.5 (2.2)	0–10	0.95^**^	0.74^*^ (0.06)
Crisis	4.1 (1.8)	0–8	0.72^*^	0.70^*^ (0.07)
Thinking	3.3 (2.2)	0–8	0.94^**^	0.78^**^ (0.06)

* *p < 0.01*.

** *p < 0.001*.

#### Validity

As illustrated in Table 2, the AUC for the discrimination of group membership showed large effect sizes for both the RoSP SPJ [AUC = 0.83, 95% CI [0.73, 0.93], *p* < 0.001] and RoSP scores [AUC = 0.80, 95% CI [0.69, 0.91], *p* < 0.001]. Three of the sub-scales (Current Clinical, Current Crisis, and Current Thinking) significantly discriminated between those who completed suicide and those who died from natural causes/accidental death with large effect sizes.

Data from the individual items of the RoSP are presented in Table 3. It is clear that not all items of the RoSP had predictive power in this sample. Indeed, some of the items appear to be “anti-predictive” (though not significantly so). For example, people who had completed suicide scored lower on the item “History of Mental Disorder.”

**Table 3:** Mean RoSP scores and inferential statistics for the patients grouped into suicide vs. control for study 1.

	**Control**	**Suicide**	***t***	***p***	**Hedges *G***
Suicide	0.63	1.29	3.42	< 0.001	0.83
Self-injury	0.57	0.66	0.45	0.33	0.11
Violence	0.60	0.72	0.61	0.28	0.15
Mental disorder	1.80	1.61	−1.33	0.30	−0.26
High risk group	0.63	1.11	2.21	0.02	0.53
Personality Dis	0.34	0.38	0.23	0.41	0.05
Symp. Depression	0.67	1.46	4.74	< 0.001	1.17
Substance misuse	0.63	1.41	3.69	< 0.001	0.89
Symp.of MD	0.77	0.84	0.33	0.37	0.08
Treatment compl.	1.03	1.43	2.03	0.03	0.50
Loss	0.46	0.72	1.21	0.12	0.30
Health	1.03	0.79	−1.28	0.27	−0.31
Relationships	0.87	1.54	4.28	< 0.001	1.04
Employment	0.80	1.11	1.60	0.06	0.39
Law	0.20	0.47	1.85	0.04	0.45
Support	0.90	1.03	0.80	0.22	0.19
Hopelessness	0.42	0.89	2.31	0.01	0.57
Anger	0.67	0.74	0.36	0.36	0.09
Ideation	0.11	1.08	6.59	< 0.001	1.65
Preparation	0.04	0.57	3.76	< 0.001	0.94

### Study 2: Inpatients

#### Reliability

Ten cases (five men) were evaluated independently by two raters and the ICCs are shown in Table 4. All scales and subscales exhibit good to excellent reliability.

**Table 4:** Descriptive and inferential statistics for the RoSP in study 2.

	**Mean (SD)**	**Range**	**ICC**	**AUC Self-injury**	**AUC Suicide attempts**	**Rho Self-injury**	**Rho Suicide attempts**
SPJ	3.4 (1.09)	2–5	0.93^**^	0.81^**^	0.80^**^	0.61^**^	0.56^**^
Total score	23.0 (4.8)	13–34	0.96^**^	0.73^*^	0.60	0.35^*^	0.19
History	7.2 (2.2)	3–14	0.96^**^	0.67^*^	0.60	0.23	0.15
Clinical	7.4 (1.7)	2–10	0.93^**^	0.57	0.44	0.02	−0.07
Crisis	3.1 (1.8)	0–7	0.79^*^	0.46	0.41	−0.06	−0.16
Thinking	5.1 (2.7)	0–10	0.86^**^	0.75^**^	0.71^**^	0.50^**^	0.39^**^

* *p < 0.01*,

** *p < 0.001*.

#### Validity

Approximately two-thirds (68.3%) of the service users recorded at least one incident of self-injury in the 3-month time period following admission to hospital, with some harming themselves on many occasions (Mean = 18.7, Median = 5.0, Range 0–139). Considering only the instances that were judged to have suicidal intent, 47.5% of the service users had at least one incident (Mean = 3.42, Median = 0, Range 0–31).

Table 4 illustrates the results relating to the efficacy of RoSP. Evaluation of any self-injury produced large effect sizes for the RoSP SPJ [AUC = 0.81, 95% CI [0.69, 0.93], *p* < 0.001] and for the RoSP scores [AUC = 0.73, 95% CI [0.57, 0.87], *p* < 0.01]. When considering instances that were coded as suicide attempts, the RoSP SPJ was a strong predictor [AUC = 0.80, 95% CI [0.69, 0.91], *p* < 0.01] while the RoSP scores were not a significant predictor [AUC = 0.60, 95% CI [0.44, 0.73], ns]. There were no completed suicides during this period of study.

An examination of the subscales of the RoSP (Table 4) shows that the Current Thinking scale was highly associated (large effect sizes) with both outcome measures (incidents of self-injury and incidents of attempted suicide), whilst the History scale had some moderate associations with self-injury. However, the Current Clinical and Current Crisis scales had little ability to discriminate those service users that went on to engage in self-injury behaviors in this context. For completeness, we present data from the individual RoSP items in Table 5. Again, it should be noted that not all items are predictive of self-injury in this sample, with some items (e.g., Treatment Compliance) appearing to be somewhat anti-predictive of self-injury.

**Table 5:** Mean RoSP scores and inferential statistics for the patients grouped as to whether they have an instance of deliberate self-injury during the study period.

	**No Self-injury**	**Self-injury**	***t***	***p***	**Hedges *G***
Suicide	1.04	1.85	4.66	< 0.001	1.26
Self-injury	1.05	1.88	5.22	< 0.001	1.42
Violence^a^	1.83	1.89	0.43	0.34	0.16
Mental disorder	1.57	1.79	0.44	0.33	0.12
High risk group^b^	1.23	1.15	−0.38	0.71	−0.10
Personality Dis.	1.90	1.97	0.02	0.49	0.32
Symp. Depression	0.40	1.37	4.66	< 0.001	1.29
Substance misuse	1.17	1.47	1.00	0.33	0.36
Symp of MD^a^	1.24	1.36	0.99	0.16	0.09
Treatment compl.	1.86	1.58	−1.78	0.08	−0.48
Loss	0.43	0.68	1.24	0.11	0.33
Health	0.38	0.49	0.58	0.29	0.16
Relationships	0.71	0.56	−0.90	0.37	−0.24
Employment	0.33	0.48	0.71	0.24	0.19
Law	0.90	1.03	0.51	0.31	0.14
Support	1.05	1.23	0.96	0.17	0.26
Hopelessness	0.57	1.36	3.51	< 0.001	1.00
Anger	1.38	1.58	1.07	0.15	0.29
Ideation	0.38	1.25	4.00	< 0.001	1.08
Preparation	0.05	0.87	4.53	< 0.001	1.22

a *These items changed substantially when RoSP 2.0 was updated to RoSP 3.0. Only data from RoSP 3.0 is presented here*.

b *This item underwent minor modifications when RoSP 2.0 was updated to RoSP 3.0. Data from both are combined in this analysis*.

## Discussion

Across two studies including very different mental health populations and different settings, we have shown that the RoSP can be reliably evaluated and is a valid indicator of serious self-injurious behavior, suicide attempts, and completed suicide.

There are already many instruments that could be used to make a prediction about the likelihood of future suicide attempts [e.g., (11, 26–28)], but analyses of these instruments have shown that they have limited clinical use, mainly due to the low prevalence rates of the behavior being predicted (9, 29). Instead, it is suggested that each individual being assessed for suicide risk needs a careful examination of their clinical needs and psychosocial vulnerabilities, across a range of different factors that have been found to be important to suicide risk. Therefore, the aim of RoSP was to produce a systematic guide to the evaluation of the needs of the individual across four domains (History, Current Clinical, Current Crisis, and Current Thinking) that clinicians could use to structure their clinical evaluations and use across a range of clinical groups and different contexts. As such, the items were chosen with safety-planning, clinical intervention, and risk reduction and management as the over-arching aim. Hence, the items needed to have reasonable “availability” to clinicians and, ideally, should be amenable to change (i.e., capable of demonstrating potential “treatment responsiveness” or response to intervention). Our demonstration in the present paper that the RoSP's psychometric properties are in-line with similar SPJs used for the assessment of other adverse events, such as violence to others, demonstrates that the RoSP has reliability and validity. Importantly, our aim has not been to attempt to make comparisons with other instruments used for the evaluation of suicide risk (especially so-called actuarial instruments). This is because to attempt to do so would not be comparing like with like, given that the key aim of the RoSP is to develop a SPJ tool that is consistent with NICE guidelines, and which attempts to focus the mind of the evaluator on safety planning and intervention, rather than solely on accurate prediction. Indeed, with regards to the in-patient sample, the Structured Professional Judgment made after completing the RoSP, produced more accurate predictions of future suicidal behavior compared to the total RoSP score, as would be predicted. This finding places further emphasis on the point that the RoSP should not be used as a predictive or actuarial tool, but as a method of understanding risk and facilitating appropriate safety planning.

Looking across the two studies, some individual items of the RoSP were effective across both studies, such as a history of previous suicide attempts, current symptoms of depression, suicidal ideation, and preparatory activity. All of these factors are very well-established in the research literature on suicide completion (29) and our studies confirm their utility across these two very different service contexts. Other items or even subscales, for example Substance Use Problems, were related to self-injurious behavior in one context (community sample), but not in the other (in-patient sample). These differences may be due to the different contexts of the two samples. The in-patient sample had severely restricted access to alcohol and other substances of abuse within secure service provision, and therefore the power of this risk factor to bring about adverse outcome would have been severely restricted. Similarly, the item “Personality Disorder” was not predictive in sample two (in-patient sample). This is due to a lack of variance, where all patients within this sample had a diagnosis of personality disorder. We, therefore, argue that the evaluation of Personality Disorder in suicide risk on the RoSP should continue to be included, given that this item will most probably be of importance to clinical evaluation in samples with more mixed diagnostic characteristics. It is to be hoped that this research article will serve as a trigger to other researchers to evaluate the RoSP as an aid to clinical evaluation of suicide within other clinical settings and services, across different countries and cultures, and it will be expected that different combinations of risk factors and sub-scales will be more powerfully associated with adverse clinical outcomes in different cohorts of service-users.

Finally, some items did not appear to have any value in either setting (e.g., violence). One might be tempted to drop such an item to make a more streamlined assessment, or to replace it with another risk factor that is associated with completed suicide. Further, by giving stronger “weighting” to items that are more strongly associated with suicide one could improve on the ability of the RoSP to identify those at risk of completed suicide. However, we emphasize that predictive validity is not the major aim of the RoSP. Rather, intervention and enhanced safety-planning is the ultimate goal, ensuring that a comprehensive assessment is completed across all necessary clinical and psychosocial areas of potential importance to the individual clinical formulation. Thus, we chose items with a good evidence-base for association with suicide risk, that were likely to be easily available to a clinician, and which could be targeted for intervention. We maintain that across other contexts, (e.g., prison populations; young men from disadvantaged areas) these items will probably prove their worth and we do not want, at this juncture, to amend the RoSP prematurely.

### Limitations and Strengths

There are a number of limitations to our current studies. The investigation of suicidal behavior poses significant ethical issues and we were only able to gather modest sample sizes. Fortunately, the effects sizes produced by the RoSP were very large and therefore even these modest samples were able to give highly significant results. However, the modest sample sizes have not allowed us to look at other important factors that might moderate the effectiveness of the RoSP, such as the effects of gender or the interval between assessment and outcome. Future studies are needed to investigate these factors, and to expand the evidence-base to other clinical settings such as emergency departments, child and adolescent mental health services, older adult services, prisons, and to other countries and cultures.

We also acknowledge that while our research has shown that the RoSP is predictive of suicidal behaviors, if it is used simply to categorize people in settings with low base-rates of suicide, its predictive value will remain low due to the low base rate of this behavior (29). Further, many people who die by suicide are not known to mental health services (30). Thus, our approach of increasing the quality of assessment and safety-planning of service users through RoSP, ensuring that this is adherent to NICE guidelines, and trying to focus on psychosocial needs as well as clinical presentation, has to also be accompanied by population-based strategies to suicide prevention.

A strength of RoSP is that it was developed and refined while being used in an actual service where assessment of suicide risk was one of the paramount issues in the management of these patients. The items and their scoring were therefore tested and altered by this “real-world” application of the instrument where issues, such as what information is available from clinical notes and interviews, place constraints on the usability of an instrument, even if it has a good research pedigree (31, 32).

### Conclusion

In conclusion, we designed RoSP from factors known to be associated with suicide and attempted suicide. We have shown that it can produce reliable and valid clinical judgments of suicide risk across two very different clinical settings: a community sample of people known to mental health services; and a sample of in-patients in low secure provision all of whom had a diagnosis of personality disorder. The structured professional judgement of suicide risk produced by the RoSP was related to suicidal behaviors in both clinical contexts with an efficacy equal or superior to other well-established structured professional judgment schemes for the prediction of violence to others. As such, we believe that these data provide the first step in the validation of the use of structured professional judgement methodology for suicide risk evaluation and safety-planning. We believe that the RoSP meets NICE (12) guidelines that:

“All people who have self-harmed should be offered an assessment of needs, which should be comprehensive and include evaluation of the social, psychological, and motivational factors specific to the act of self-harm, current suicidal intent and hopelessness, as well as a full mental health and social needs assessment.” (1.4.1.5 p.6).

RoSP can therefore provide a structure for meeting NICE guidelines for suicide prevention. Further studies are now needed to see if the scientific and clinical implementation of RoSP produces improved safety-planning and improved outcomes for service users across a range of clinical services and cross-culturally.

## Data Availability Statement

The raw data supporting the conclusions of this article will be made available by the authors, without undue reservation.

## Ethics Statement

The studies involving human participants were reviewed and approved by National Health Service Research Ethics Committee (15/EM/0044) and National Health Service Research Ethics Committee (14/EM/1178). Written informed consent for participation was not required for this study in accordance with the national legislation and the institutional requirements.

## Author Contributions

NG was a co-author of the RoSP scheme, helped design both experiments, led the data collection and project management, and participated in writing the manuscript. AJ advised on analysis of the data and commented on drafts of the manuscript. AM and SR completed the RoSP assessments in both study 1 and study 2 and commented on drafts of the manuscript. JK completed the RoSP assessments in study 2 and helped in writing the manuscript and with amendments to the RoSP scheme. RS was a co-author of the RoSP scheme, helped design both experiments, analyzed the data, and wrote the initial draft of the manuscript. All authors contributed to the article and approved the submitted version.

## Conflict of Interest

NG and RS are authors of the Risk of Suicide Protocol. At present, they received no royalties or payments for the use of this instrument. The remaining authors declare that the research was conducted in the absence of any commercial or financial relationships that could be construed as a potential conflict of interest.

## Footnotes

^1^The term “current” is meant to refer to the recent past and/or whether the effects of this risk factor, or area of clinical need, are still having an impact on the person. For example, the item “Recent Loss of Significant Other” could still be regarded as “present” if the person is still experiencing active psychological distress or grief from the loss, even if this significant other died several years ago.
